# Effectiveness of soil remediation intervention in abandoned used lead acid battery (ULAB) recycling sites to reduce lead exposure among the children

**DOI:** 10.1016/j.mex.2024.102772

**Published:** 2024-05-29

**Authors:** Mahbubur Rahman, Jesmin Sultana, Shaikh Sharif Hasan, Syeda Nurunnahar, Musa Baker, Rubhana Raqib, Syed Moshfiqur Rahman, Maria Kippler, Sarker Masud Parvez

**Affiliations:** a Global Health and Migration Unit, Department of Women’s and Children’s Health, Uppsala University, Sweden; b Environmental Health and WASH, Health System and Population Studies Division, icddr,b, Mohakhali, Dhaka, 1212, Bangladesh; c Nutrition Research Division, icddr,b, Mohakhali, Dhaka, 1212, Bangladesh; d Institute of Environmental Medicine, Karolinska Institutet, 17177, Stockholm, Sweden; e Children's Health and Environment Program, Child Health Research Centre, The University of Queensland, South Brisbane, QLD 4101, Australia

**Keywords:** Heavy meta, Intervention, Biomarker, Quasi-experimental, Bangladesh

## Abstract

Lead (Pb) is a neurotoxin, and children are vulnerable due to their evolving physiology and high-risk behaviours. Soil remediation interventions have proven effective in reducing Pb exposure. The primary objective is to measure the effectiveness of soil remediation at abandoned used lead acid battery (ULAB) recycling sites, nearby household cleaning, and community awareness in reducing blood lead levels (BLLs) in children. Additionally, this study aims to examine associations of Pb exposure with hematological, cardiovascular, renal, immunological, and endocrinological parameters in children aged 0–12 years. This study employs a quasi-experimental design, with abandoned ULAB sites as intervention sites and two control sites in Bangladesh. The intervention includes soil remediation coupled with community education. Data will be collected prior to the intervention and at a 12-month follow-up, including a comprehensive Pb exposure survey and collect environmental, turmeric samples, and blood from the child. Pb concentrations in environmental samples and turmeric samples will be determined using XRF analyser. Child BLL will be measured using Graphite Furnace Atomic Absorption Spectrometry (GF-AAS) and proposed biochemical parameters will be analysed using routine laboratory methods. This study could provide valuable insights for designing targeted interventions in similar settings and mitigating exposure to Pb.

## Graphical abstract

**Figure:**
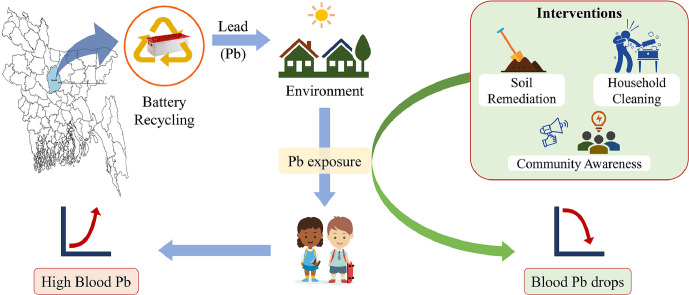

Specifications table

**Table:** 

Subject area:	Environmental Science
More specific subject area:	*Lead (Pb) toxicity*
Name of your protocol:	Effectiveness of soil remediation intervention in abandoned ULAB recycling sites to reduce lead exposure among the children
Reagents/tools:	*Not applicable (NA)*
Experimental design:	*Quasi-experimental*
Trial registration:	*NA*
Ethics:	*The study protocol has received approval from the Institutional Review Boards of the International centre for Diarrheal Disease Research, Bangladesh (icddr,b). Informed written consent will be taken from the primary caregiver of all participating children.*
Value of the Protocol:	*• Employing an experimental design to evaluate how soil remediation intervention impacts child blood Pb level.* *• Implementing a comprehensive measurement of exposures, covariates, and diverse health outcomes.* *• Inclusion of double control arm to enhance the precision of estimates and better understand the exposure-outcome relationship.*

## Description of the protocol

### Background

Lead (Pb) is a highly toxic environmental pollutant that is detrimental to the environment and human health [1]. Exposure to Pb can produce alterations in various physiological functions of the body and has been associated with many adverse health outcomes, including neurological, respiratory, kidney, and cardiovascular disorders [[2], [3], [4], [5], [6]]. Furthermore, Pb disturbs the oxidant–antioxidant balance, induces inflammatory responses in various organs and modulates the immune system. Pb is most detrimental to children due to their developing physiology and exposure routes, including placental transfer, breastfeeding, and frequent hand-to-mouth behavior [3,7]. Evidence suggests that there is no threshold for Pb-induced neurodevelopmental effects [8,9]. It is therefore, essential to reduce the use and spread of Pb in the environment.

Recycling of used Pb acid batteries (ULABs) has been identified as an important source of Pb exposure, among workers and people living near recycling facilities [10]. Data from a review of studies of battery workers from 37 low-and middle-income countries (LMICs) revealed that the average blood lead level (BLL) of young children living close to battery manufacturing and recycling facilities was 19 µg/dL [11]. In Bangladesh, there are more than 10,000 battery recycling and recharging facilities, employing more than 15,000 adults and 5000 children (5–17 years old), and the low labor and processing costs and high- profit margins have resulted in an unregulated battery recycling market is growing rapidly [10]. Using primitive and extremely polluting smelting methods, such as smelting in open pits, and untreated waste disposal in the environment, has been associated with adverse effects of Pb poisoning in the communities around numerous informal ULAB recycling sites [10,12]. Also, several years after recycling operations have stopped at ULAB recycling sites, the soil remains contaminated, and the risk of Pb exposure is still high [13].

Soil remediation intervention has been proven to be a successful approach to lower soil Pb levels, hence reducing Pb exposure in the nearby population [13,14]. There are several different soil remediation techniques. Studies in Bangladesh has reported that by adopting the soil scrapping, removal, and capping approach, soil Pb levels were reduced by more than 96% (1400 at baseline to 55 mg/kg), and at the 14-month follow-up, the median BLL of the local children had decreased from 22.6 µg/dL to 14.8 µg/dL [13].

### Study objectives

Given the likely long-term adverse health consequences from Pb exposure, the scientific community and developmental agencies would benefit from robust evidence of an intervention targeted at the household level. The primary scientific objectives of this trail are to, 1) measure the effectiveness of the soil remediation of an abandoned ULAB recycling site, nearby household cleaning, and a community awareness intervention in reducing BLL in children aged 0 to 12 years; 2) assess if the community awareness intervention has improved Pb exposure prevention knowledge among the caregivers of participating children; 3) determine the characteristics (behavioural/nutritional) associated with BLL among the participating children. The secondary objective includes 1) measure the association between Pb exposure and hematological parameters related to hemoglobin (Hb) synthesis in children (0–12 years) (i.e., Hb, serum ferritin, vitamin B12, and folate); 2) measure the longitudinal association between Pb exposure and cardiovascular risk factors (blood pressure, lipid profiles, pro-inflammatory markers, endothelial adhesion molecules) and insulin-like growth factor 1 (IGF-1) among 0–12 years old children; 3) determine the concentrations of other toxic metals in the blood (Cd, Cr, Hg) and urine (As) of the participating children and in environmental samples (soil, water) from the childrens home and surrounding environment; and 4) investigate the acceptability of the intervention by the targeted households and community.

## Methods

### Study design

**Fig. 1:**
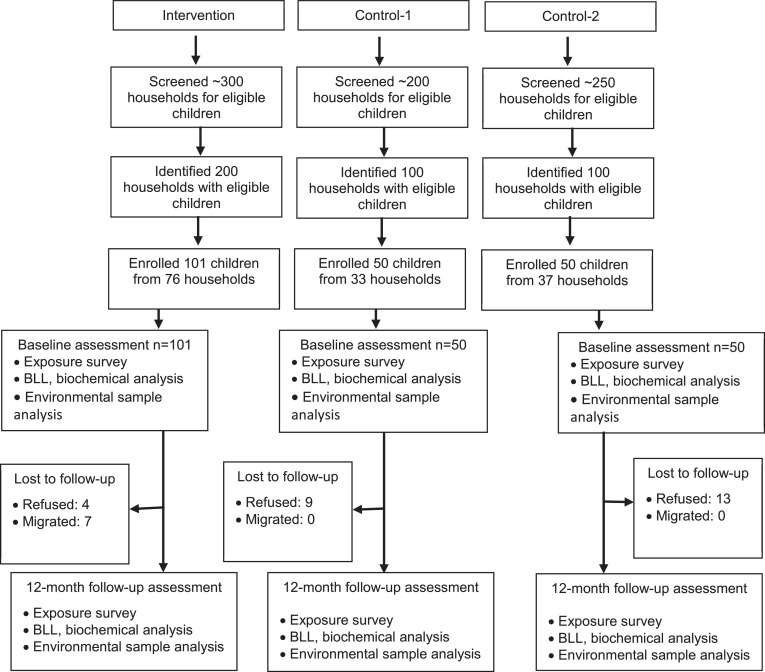
Study enrolment flowchart.

To achieve the study objectives, we will conduct a pilot study employing a quasi-experimental research design (Fig. 1). The proposed study design includes an intervention arm (*n* = 100), a small-sized control arm (*n* = 50), and a second control arm (*n* = 50) comprised of the general population from the same geographical region who have never been exposed to ULAB recycling. With no ULAB Pb exposure, the second control arm will serve as a reference group for BLLs from the general population. Thus, the purpose of the second control arm is to evaluate the intervention's efficacy in lowering BLLs to those found in the general population.

### Study site selection

**Fig. 2:**
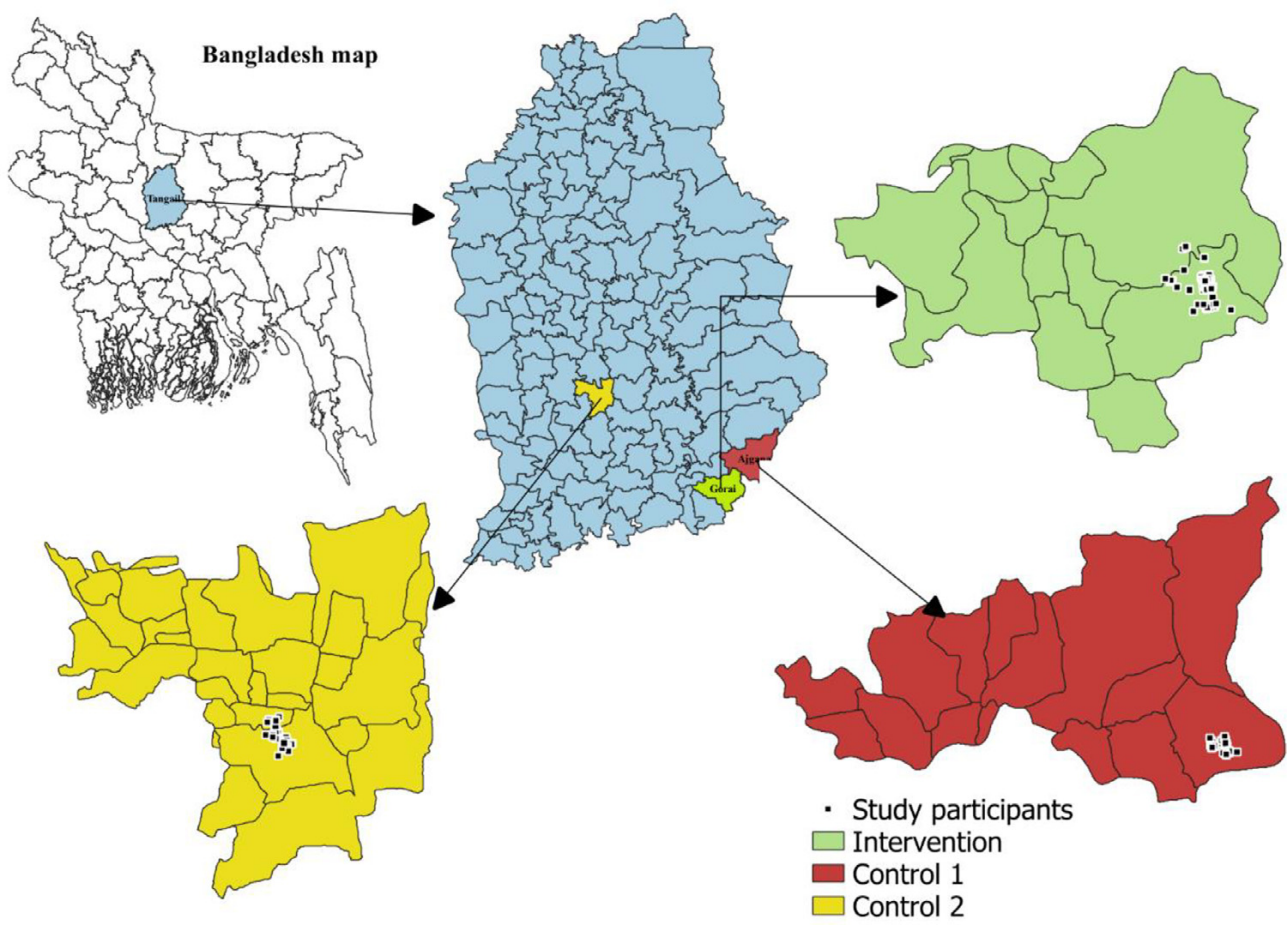
Study site.

The study will be conducted in Tangail district of Bangladesh. The intervention location is in the sub-district of Mirzapur, where the ULAB recycling activity was shut down more than a year ago (Fig. 2). Thus, this site was identified as an ideal location for a demonstration remediation project.

For control area 1, the team will select an abandoned ULAB recycling site located around 5 km away from the intervention site, which has been matched with the intervention arm in terms of geographical location and ULAB exposure (i.e., abandoned time). Control area 2 will be the nearest eligible village within the same geographical region without any ULAB recycling within a 5 km radius (Fig. 2). The selection of 5 km radius aligns with WHO recommendations, as Pb contamination can spread up to 1.7 miles (2.7 km) from a ULAB recycling site [15]. Also, establishing this buffer zone, may reduce cross-contamination for both controls.

### Study population eligibility and recruitment

The study population comprises children aged 0 to 12 years who have resided within a 750-meter radius of the selected ULAB recycling sites for at least six months. Target children are only eligible to participate if the parents/guardians intend to stay in the study area for 18 months, starting from the time of household listing. Critically ill children will be excluded from participation. The subject population will include both boys and girls, and no child will be excluded based on race, ethnicity, language, or literacy.

Before conducting the baseline survey, a trained field team will screen every household within a 750-meter radius of the abandoned ULAB recycling site and make a list of eligible children for the intervention and the first control area. For the second control area, the team will compile a list of eligible children within the 750-meter radius of the locally identified middle point of that village. Finally, the investigator will randomly select the desired number of children from two different age strata (0–5 and 6–12 years), considering a 1:1 ratio from each study site. In case of unavailability of children for the specific age group, the study investigator will replace them with children from the other age group.

### Intervention implementation

The intervention implemented by Dhaka University, involves the following components both at the abandoned site and within each household at the site: i) community education about Pb hazards and exposure routes, ii) removal of Pb contaminated waste, iii) in-situ dilution of low-level contaminated soils, iv) encapsulation or disposal of highly contaminated soil to break exposure pathways, and v) clean-up of contaminated house interiors with the help and approval of household members and landlords.

The goal of the remediation is to ensure that the soil Pb doesn’t exceed the US EPA's recommended limit for residential soil, which is 400 mg/kg. Where soil contamination is less severe, the implementation team will employ in-situ dilution method, involving mixing of clean soil with contaminated soil until concentrations fall below 400 mg/kg. For high soil-Pb concentrations exceeding 2000 mg/kg, the contaminated soil will be scraped and deposited in a dedicated containment structure, either on-site or in a nearby landfill. These measures aimed to prevent new exposures during future development or alternative land uses.

### Data collection

The evaluation activities will be carried out at two time points: at the baseline assessment stage, prior to the implementation of the intervention, and at the 12-month follow-up after the intervention. This study will be conducted by a team comprising medical professionals, epidemiologists, social scientists, environmental scientists, and laboratory technicians. The data collection activities will include anthropometric and blood pressure measurements, a dietary diversity assessment, an exposure assessment, and the collection of biological and environmental samples. This study did not account for seasonal variability during data collection. Data collection tools will be adapted from a previous study conducted in a similar setting, and piloted and revised to make the tools context-specific [16]. Following recruitment, the field team will receive training on the piloted exposure assessment questionnaire through facilitated discussion, role play, field tests, and the use of a tablet platform before beginning data collection. The trained field workers will visit the sites, approach eligible participants, and explain the study in detail before data collection. All the data will be collected electronically.

### Anthropometry and blood pressure measurement

Child weight, height or length, waist circumference, hip circumference, and mid upper arm circumference (MUAC) will be measured at baseline. The child will be instructed to wear light clothing during the measurement. The field team will be trained and standardized according to FANTA and WHO guidelines [17]. The instruments will be calibrated every morning before fieldwork and a logbook will be maintained. In addition, a trained physician will use an appropriately sized cuff and calibrated instrument to measure a child's blood pressure in their own setting. An average of three measurements will be taken for each parameter.

### Dietary diversity assessment

Earlier studies have shown that ultra-processed food can be contaminated with Pb [18]. The team will therefore, measure dietary diversity by considering 24-hour recall and consumed food processing status, including unprocessed or minimally processed, processed ingredients, processed, and ultra-processed [19]. The level of nutritional adequacy and risk of malnutrition can be estimated using the individual's dietary diversity score and frequency of ultra-processed food intake.

### Exposure assessment

To determine the risk of child Pb exposure from abandoned ULAB recycling sites and control sites, trained field research assistants will administer a Pb knowledge and exposure assessment questionnaire to the child's caregivers during the survey. Child behavior, including crawling across a floor, mouthing toys or other objects, and frequent hand-to-mouth activities, increases the exposure. Thus, the exposure assessment questionnaire will record the caregiver-reported daily activities of the child, such as soil ingestion habits, food habits (polished turmeric intake), use of glazed or painted ceramic utensils for eating and/or cooking, use of cosmetics (i.e., nail polish, kohl/kajol), intake of traditional medicine, and the habit of playing near or inside ULAB recycling sites. The team will also collect data on current or past home-based or other Pb recycling activities by family members, proximity to current or past recycling activity, household cleaning method and frequency, type of cookware used by the households, type of turmeric used by the households for cooking, availability of paint chips in the household, and household characteristics at each participating household.

### Biological sample collection and biomarkers analysis

A team of trained medical technologists and field assistants will collect 6 mL of blood through venipuncture from all enrolled children using trace metal-free certified needles and tubes during baseline and 12-months post-intervention follow-up assessments. All the samples will be transported to the icddr,b laboratory within 4–6 h of collection in a cooler box. Aliquots of whole blood will be stored in −80 °C freezers for metal analysis. In addition, a medical technologist will perform centrifugation (894 g relative centrifugal force) for 15 min at ambient temperature for serum separation, and aliquots will be stored in *a* − 80 °C freezer at icddr,b. The details of the biomarkers analyses are given below (Table 1):

**Table 1:** Study outcome indicators and measurement tools.

Primary outcome		
**Sample analysed**	**Indicator**	**Measurement tool**
**Metal analysis**		
Whole blood	Blood Pb level	Graphite furnace atomic absorption spectrometry (GF-AAS)
**Secondary outcomes**		
**Targeted system & sample analysed**	**Indicator**	**Measurement tool**
**Cardiovascular system**		
Serum	TG, Tc, LDL, HDL	Evolution 3000 semi-automated biochemistry analyzer
Serum	Lp-PLA_2_	PLAC®Test (diaDexus Inc, San Francisco, CA, USA)
**Renal system**		
Serum	creatinine and eGFR	semi-auto electrolyte analyser (biolyte2000, bio-care corporation, taiwan)
**hematological system**		
Serum/Whole blood	CBC, Hb, Ferritin	Automated analyser (COBAS e601)
**Immune system**		
Serum	Pro-inflammatory role of cytokines (IL-6 and TNF-α)	Enzyme-linked immunosorbent assay
	Endothelial Adhesion Molecule ,intercellular adhesion molecule-1 (ICAM-1), vascular cell adhesion molecule-1 (VCAM-1), and E-selectin	Enzyme-linked immunosorbent assay
**Endocrine system**		
Serum	HbA1c, Insulin-like growth factor 1 (IGF-1)	Enzyme-linked immunosorbent assay

Field workers will instruct and collect 50 mL of spot urine in a falcon tube which will thereafter be transported to the icddr,b laboratory at 2–8 °C within 6 h of collection. The samples will be stored in −80 freezer for future metal analysis, such as arsenic (As) once additional funding has been secured.

### Environmental sample collection and analysis

A trained team will collect soil and dust samples from various locations, including ULAB recycling site, child's play area, courtyard, living dwelling floor, and roadside. To collect soil, field workers will identify five different locations in the child's play area and courtyard (four corners and the center point) and collect approximately five grams of soil from each point, from the top five centimetre of the soil [20]. 20–25 g of composite soil samples will be collected from each sampling site in a ziplock bag and transported to icddr,b for Pb concentration measurement using a hand-held XRF analyser. Dust samples will be collected from different surfaces, such as the courtyard and child's play area. Two types of dust samples will be collected: sweep dust and wipe dust. For sweep dust samples, household member will be asked to use their regular broom to sweep dust from the floor surface. A pre-cleaned paint brushes will then be used to collect the dust which will be stored in Ziploc bags. The length and width of the sleeping dwelling and the courtyard will be recorded using measuring tape to calculate the surface area of the collected sweep dust sample. For wipe dust sample collection, a pre-cleaned 10 × 10 cm frame will be placed on the sleeping dwelling floor, commonly accessed by the study child. A clean, wet tissue wipe will be placed inside the frame, and an “S”-like motion will be used to wipe the entire sample area using firm pressure. After the first wipe, the wipe tissue will be folded in half, and this folding and wiping process will be repeated four times to wipe and collect dust samples from the selected area. The collected wipe dust samples in the tissue wipe will be then transferred to a Ziplock bag and stored at icddr,b for future analysis.

The research team will also collect a drinking water sample from each household, assuming that acid inside the battery case is frequently dumped on the ground or connected directly to a drainage canal or sewer, which may result in groundwater or surface water contamination. The drinking water samples will be collected from the source using a 50 mL falcon tube. Within 6–8 h of collection, the water samples will be delivered to the icddr,b laboratory, and stored at 2–8 °C for future metal analysis. The team will collect turmeric samples from every household. Forsyth and colleagues reported that loose powdered turmeric purchased from local markets are more likely to contain Pb than packaged powdered turmeric. Similarly, polished dried turmeric roots were more likely to contain Pb than unpolished roots [21]. Each household will be asked about the type and source of turmeric they use in cooking. Then, the data collectors will request 10–15 g of the turmeric powder from the household. They will provide a pre-cleaned spoon to transfer the turmeric powder from their container to a Ziplock bag. The collected samples will be transferred to icddr,b for further analysis. In case where households possess dry turmeric roots, they will be asked to provide 3–4 roots. These dry turmeric root sample will be transported to icddr,b in a Ziplock bag. Before XRF analysis, the samples will be grounded using a mortar and pestle. In addition, the team will also collect chili powder and paint chips from every household. The team will use the same SOP for turmeric and chili powder samples. For paint chips sample collection, if visible cracks are present in the painted walls of the households, the data collectors will seek permission to use forceps and sharpies to extract the paint chips from the walls and will be stored in a Ziplock bag. To enumerate toxic metals, soil, dust, and turmeric samples will be analysed using portable XRF (X-ray fluorescence) [Olympus; DMTA-10075-01EN] at icddr,b. Prior studies identified XRF as a rapid, accurate, and efficient method to map metal-contaminated soils [22,23]. The U.S. Environmental Protection (EPA's) Method 6200 highlights using XRF to determine the types and concentrations of metals in soil and sediments. An aliquot of dust and soil samples will be stored for future analysis.

### Qualitative assessment

A research team will conduct a qualitative assessment to assess the feasibility and acceptability of Pb exposure reduction intervention from an abandoned ULAB recycling site. Based on our previous study experience, this qualitative assessment will be conducted after the 12-month follow-up assessment. This qualitative assessment will include in-depth interviews with the participating households, focus group discussions with the community members, key informant interviews with the community leaders, intervention implementers, physicians or health workers, and other key stakeholders, such as other NGO workers involved in relevant works, and food and agriculture-related organization's key staff. We aim to conduct one key informant interview (KII) with each category respondent, two focus group discussions (FGDs) with community members, and 18 in-depth interviews (IDIs) with the participating households. A trained qualitative team will conduct these qualitative assessments. However, the proposed number of qualitative assessments may vary based on the preliminary analysis and data saturation.

### Sample size calculation

The primary outcome of this study is the rate of reduction of child BLL after the intervention. The sample size was calculated using mean BLLs from an earlier study [13]. In that study, the mean BLL was reduced from 21.08 µg/dL at baseline to 15.19 µg/dL at the 14-th month follow-up (5.89 µg/dL reduction). Given that the investigator will implement the same intervention as in the prior research, anticipate a similar rate of reduction. We assume that following a 12-month intervention, the mean BLL in the control and intervention group will be 21.08 µg/dL (SD: 8.87) and 15.19 µg/dL (SD: 3.95), respectively. We have estimated that the control sample size will be 50% less than the intervention. The sample size was calculated as 50 participants in each age stratam for the intervention arm and 25 participants in the control arm, considering 80% power, 95% confidence interval, and 1.04 design effect. Additionally, the investigator increased the sample size, considering 20% lost to follow-up. Thus, this study will require a total of 100 individuals for the intervention arm and 50 participants for the control arm based on 1:1 stratified sampling for each age stratum. The team will use the same sample size from the general population for the second control arm as we did for the first control arm to get baseline BLL values for the unexposed ULAB group. In summary, this study will have a total of 200 study participants, with 100 in the intervention group and 50 in each of the first and second control groups, respectively.

### Data analysis

Data will be analysed using Stata Statistical Software: Release 13 (StataCorp LP, College Station, TX). For descriptive analysis, proportions will be calculated for binary and categorical variables, median and interquartile range (IQR) for continuous variables and summarized in descriptive tables. This study will measure the intervention's effect size and mean difference of BLLs from baseline to 12-month follow-up using the Generalized Estimating Equation (GEE) method. The investigator will adjust for the theoretical covariates of increased BLL among children to estimate the intervention's effect, such as the availability of Pb-soldered cans in the household, availability of family members who work in the Pb industry, and Pb concentration in environmental samples, and distance from the ULAB sites. The research team will compare the rate of BLL reduction in the intervention arm with the control arms. Similarly, multivariable regression analysis will be conducted to evaluate associations of Pb exposure with cardiovascular, renal, hematological, immunological, and endocrinological parameters.

For qualitative data analysis, the team will use a data organizing software, ATLAS.ti version no 5.2. They will develop a coding system that involves reading through the interviews several times and looking for similarities and differences. All the coded data will be entered into the ATLAS.ti software to identify research themes and concepts. Comparison and triangulation within themes will be conducted using the various data collection techniques.

### Ethics and dissemination

Informed written consent will be obtained from the primary caregiver of all participating children. The study protocol has been rigorously reviewed and approved by the Research Review Committee (RRC) and the Ethical Review Committee (ERC) of icddr,b. We will get approval for any additional modifications to the study protocol. The consent form will be translated into Bangla so that study participants with little or no formal education can understand it. During data storage, analysis, and dissemination, datasets will be anonymous without having personal identifiers, and we will strictly maintain the participant's privacy.

The results of the study will be shared with the Bangladeshi government's Department of Environment (DoE) and other partner organizations involved in Pb contamination and relevant environmental exposures. As appropriate, the findings will be share with medical experts and researchers. Also abstracts will be presented to the relevant national and international conferences and publish research articles in peer-reviewed journals.

## Conclusion and future work

The proposed study aims to evaluate the efficacy of a remediation intervention in lowering blood lead levels (BLLs) and to compare the findings with those of other populations. In the proposed study, we aim to enhance the external validity by incorporating two control arms. In a previous study conducted in a similar setting, researchers used a before-after design without a comparison group and measured Pb with LeadCare-II from capillary blood which is a point-of-care blood testing system known to overestimate the blood Pb levels compared to the GF-AAS [24]. To further enhance the validity, we propose random selection of participants and geographic matching of control arms to minimize bias and variability. We encountered a higher refusal rate in the previous study; to address this, we have calculated the sample size considering a 20% drop-out rate and propose enrolling children within a wider radius during blood collection from apparently healthy children. Furthermore, the outcomes reported in this study are likely objective measures. We will collect comprehensive data on potential exposures and covariates, employing a robust statistical approach to establish reliable and meaningful associations between different exposure-outcome relationships, thus enhancing the validity of our findings. Additionally, we will conduct a comprehensive health assessment by collecting biological samples, enabling exploration of various health parameters directly related to Pb exposure.

The results of this study could show how effective the remediation technique is and how it may help to reduce Pb exposure-related health outcomes. The study will also aid in identifying knowledge gaps and potential areas for additional research in Pb contamination-related soil remediation. It highlights the importance of ongoing research into the long-term performance and scalability of the tested remediation technique. Future research might build on these results to improve and optimize current techniques, addressing difficulties in different soil types and contaminated circumstances. Ultimately, the research findings will contribute to the body of knowledge in the field, guiding future research and promoting collaboration among stakeholders involved in soil remediation, thereby improving public health outcomes.

## CRediT authorship contribution statement

**Mahbubur Rahman:** Conceptualization, Data curation, Writing – original draft, Investigation, Funding acquisition, Project administration, Methodology, Resources, Supervision. **Jesmin Sultana:** Conceptualization, Data curation, Writing – review & editing, Investigation, Funding acquisition, Project administration, Methodology, Supervision. **Shaikh Sharif Hasan:** Data curation, Investigation, Writing – review & editing. **Syeda Nurunnahar:** Data curation, Investigation, Writing – review & editing. **Musa Baker:** Data curation, Investigation, Writing – review & editing. **Rubhana Raqib:** Conceptualization, Methodology, Writing – review & editing. **Syed Moshfiqur Rahman:** Conceptualization, Methodology, Writing – review & editing. **Maria Kippler:** Conceptualization, Methodology, Writing – review & editing. **Sarker Masud Parvez:** Conceptualization, Methodology, Writing – original draft, Visualization.

## Declaration of competing interest

The authors declare that they have no known competing financial interests or personal relationships that could have appeared to influence the work reported in this paper.

## Data Availability

No data was used for the research described in the article.
